# Lapatinib for treatment of advanced or metastasized breast cancer: systematic review

**DOI:** 10.1590/S1516-31802009000500009

**Published:** 2010-02-03

**Authors:** Rachel Riera, Patrícia Coelho de Soárez, Maria Eduarda dos Santos Puga, Marcos Bosi Ferraz

**Affiliations:** I MD, MSc. Physician and research assistant at Centro Paulista de Economia da Saúde (CPES), Universidade Federal de São Paulo (Unifesp), São Paulo, Brazil.; II MSc. Dental surgeon, professor and researcher at Centro Paulista de Economia da Saúde (CPES), Universidade Federal de São Paulo (Unifesp), São Paulo, Brazil.; III MSc. Librarian and research assistant at Centro Paulista de Economia da Saúde (CPES), Universidade Federal de São Paulo (Unifesp), São Paulo, Brazil.; IV MD, MSc, PhD. Physician and Director of Centro Paulista de Economia da Saúde (CPES), Universidade Federal de São Paulo (Unifesp), São Paulo, Brazil.

**Keywords:** Breast neoplasms, Review, Antineoplastic agents, Antineoplastic protocols, Receptor, erbB-2, Neoplasias de mama, Revisão, Antineoplásicos, Protocolos antineoplásicos, Receptor erbB-2

## Abstract

**CONTEXT AND OBJECTIVE:**

Around 16% to 20% of women with breast cancer have advanced, metastasized breast cancer. At this stage, the disease is treatable, but not curable. The objective here was to assess the effectiveness of lapatinib for treating patients with advanced or metastasized breast cancer.

**DESIGN AND SETTING:**

Systematic review of the literature, developed at Centro Paulista de Economia da Saúde (CPES), Universidade Federal de São Paulo (Unifesp).

**METHOD:**

Systematic review with searches in virtual databases (PubMed, Lilacs [Literatura Latino-Americana e do Caribe em Ciências da Saúde], Cochrane Library, Scirus and Web of Science) and manual search.

**RESULTS:**

Only one clinical trial that met the selection criteria was found. This study showed that lapatinib in association with capecitabine reduced the risk of cancer progression by 51% (95% confidence interval, CI: 0.34-0.71; P < 0.001), compared with capecitabine alone, without any increase in severe adverse effects.

**CONCLUSION:**

The combination of lapatinib plus capecitabine was more effective than capecitabine alone for reducing the risk of cancer progression. Further randomized clinical trials need to be carried out with the aim of assessing the effectiveness of lapatinib as monotherapy or in association for first-line or second-line treatment of advanced breast cancer.

## RESUMO

**CONTEXTO E OBJETIVO:**

Aproximadamente 16% a 20% das mulheres com câncer de mama têm doença avançada com metástases. Neste estágio, a doença é tratável, porém incurável. O objetivo foi avaliar a efetividade do lapatinib no tratamento de pacientes com câncer de mama avançado ou metastático.

**TIPO DE ESTUDO E LOCAL:**

Revisão sistemática da literatura desenvolvida no Centro Paulista de Economia da Saúde (CPES), Universidade Federal de São Paulo (Unifesp).

**MÉTODO:**

Revisão sistemática com busca em bases de dados virtuais (PubMed, Lilacs [Literatura Latino-Americana e do Caribe em Ciências da Saúde], Cochrane Library, Scirus and Web of Science) e busca manual.

**RESULTADOS:**

Foi encontrado apenas um ensaio clínico que preencheu os critérios de seleção. Este estudo mostrou que o lapatinib em associação com a capecitabina reduziu em 51% o risco de progressão da doença (intervalo de confiança, IC 95%: 0,34-0,71; P < 0,001) quando comparado com a capecitabina isolada, sem aumento de efeitos adversos graves.

**CONCLUSÃO:**

A combinação de lapatinib e capecitabina foi mais efetiva do que a capecitabina isolada na redução do risco de progressão da doença. Ensaios clínicos aleatórios devem ser realizados com o objetivo de avaliar a efetividade do lapatinib como monoterapia ou em associação no tratamento primário ou secundário do câncer de mama avançado.

## INTRODUCTION

Breast cancer is the most common type of cancer found in women and is the main cause of cancer deaths among women.^1^ In 2000, more than one million new cases of breast cancer were reported, and approximately 373,000 deaths worldwide were attributed to this.^1^

While many cases of breast cancer in the early stages are successfully treated, it is considered incurable when there is recurrence of the disease with metastasis. Nevertheless, such situations are treatable.^2^

Advanced or metastasized breast cancer is defined as a clinical stage that corresponds to phases III and IV of the cancer, based on the tumor itself, lymph node involvement and metastases.^3^

Approximately 16% to 20% of women with breast cancer have advanced, metastasized breast cancer, and 50% of early-stage breast cancer ultimately develops to metastasized breast cancer.^4^ Some women with metastasized breast cancer live for many years, but the average survival upon diagnosis ranges from 18 to 24 months.^3^^,^^5^ Women with advanced or metastasized breast cancer that overexpresses epidermal growth factor receptors (EGFR) have a worse prognosis, with a high risk of recurrence after the primary treatment and a 50% reduction in mean survival.^4^^,^^5^^,^^6^

The treatment objective in cases of advanced or metastasized breast cancer is to control the symptoms and prolong life, given that such cases are considered incurable and that first-line surgery will have been ineffective.^3^^,^^4^^,^^5^^,^^6^^,^^7^ Although there is no evidence from randomized studies comparing chemotherapy with observation alone, among women with this type of breast cancer, it is widely accepted that these patients should receive some type of systematic treatment at some point during the illness.^8^

In advanced cases, the therapies usually include prescription of anthracycline or a combination of cyclophosphamide, methotrexate and fluorouracil.^9^^,^^10^ The guidelines published by the National Institute for Health and Clinical Excellence (NICE) also include capecitabine, vinorelbine, docetaxel, paclitaxel, gemcitabine and trastuzumab.^4^^,^^11^^,^^12^^,^^13^^,^^14^

Considering that women with breast cancer with overexpression of EGFR are at greater risk of cancer progression, therapeutic strategies have been developed to block the activity of these receptors and to improve the response to treatment.^15^^,^^16^

The EGFR family includes four subtypes: HER-1 (human epidermal receptor), HER-2, HER-3 and HER-4.^17^^,^^18^^,^^19^ Overexpression of HER-1 occurs in approximately 27%-30% of breast cancer cases, while overexpression of HER-2 is recorded in 20%-25% of the 1.5 million new breast cancer cases diagnosed annually worldwide.^17^^,^^18^

These receptors do not stay put in a given spot on the cell membrane, and the extracellular domains of pairs of receptors attach to each other via a connection that forms a dimer. These dimers may be homodimers (i.e. two identical receptors, such as HER-1 and HER-1, etc.) or heterodimers (i.e. different receptors, such as HER-1 and HER-2, etc.).^17^^,^^18^^,^^19^^,^^20^^,^^21^ After dimerization, the intracellular tyrosine kinase domain is activated, thus stimulating cell proliferation.^17^

Lapatinib (GW572016/Tykerb; GlaxoSmithKline, Research Triangle Park, North Carolina, United States) is an oral inhibitor of HER-1 and HER-2 receptors that attaches to the intracellular tyrosine kinase domain, thereby blocking the growth of tumor cells.^17^^,^^22^ It presents some theoretical advantages over monoclonal antibodies that block the intracellular domain of the HER-2 receptor (e.g. trastuzumab, Herceptin; Genentech, Inc., South San Francisco, California, United States).^6^

Inhibition of a single receptor subtype cannot be as effective as double inhibition of the heterodimers containing HER-1 and HER-2.^23^ Moreover, there are truncated receptors that do not have extracellular domains and that are not recognized by the antibodies that connect to this domain. In fact, a truncated form of HER-2 known as p95 that has much more tyrosine kinase activity than other forms of HER-2 has been documented.^6^

Lapatinib has been effective in inhibiting p95HER-2 phosphorylation in BT474 cells and in tumor xenografts. On the other hand, trastuzumab does not bind to or inhibit the p95HER-2 receptor, which suggests that resistance to trastuzumab can be measured through the overexpression of p95HER-2 receptors during cancer progression.^24^ Overexpression of p95HER-2 receptors has also been observed as an independent prognostic factor for breast cancer cases, thus defining a group of patients with increased expression of HER-2 that demonstrates a shorter cancer-free survival period.^25^ In practice, in cases of metastasis, resistance to trastuzumab occasionally develops, in addition to recurrence after adjuvant therapy.^26^^,^^27^^,^^28^^,^^29^^,^^30^

Given the theoretical benefits of lapatinib, taking into account the prevalence of this type of cancer and the difficulty in managing it, a systematic review is necessary in order to map out the evidence available, prove the effectiveness of this medication and, thus, provide support for its use in treating advanced or metastasized breast cancer.

## OBJECTIVE

To assess the effectiveness of lapatinib as monotherapy or in association with another drug during first-line or second-line treatment of patients with advanced or metastasized breast cancer.

## MATERIAL AND METHODS

### 1. Search strategy

A systematic review of the literature was conducted from the following sources: a) virtual search: Cochrane Library, PubMed, Embase, Lilacs (Literatura Latino-Americana e do Caribe em Ciências da Saúde), Web of Science and Scirus; b) manual search: search of the references listed in articles and congress abstracts, contacts with authors and contacts with the pharmaceutical industry. The search strategy used for each virtual database is available in Table 1.

### 2. Study selection criteria

The criteria for study selection were that they should be randomized clinical trials assessing the effectiveness of lapatinib as monotherapy or in association with another drug, compared with placebo or another intervention or treatment for women with advanced or metastasized breast cancer, as a first-line or second-line treatment.

All abstracts from the 689 articles that were found were evaluated to select the likely clinical studies of interest. When in doubt, the article was evaluated in its entirety.

### 3. Assessment of study methodological quality

The following evaluation scales were used: a) Jadad, ranging from 0 to 5, in which higher ratings signified better methodological quality of implementation of the study; and b) the Cochrane Collaboration Handbook.^31^^,^^32^

## RESULTS

Three papers were selected, which each reported on a single randomized clinical trial that met the selection criteria.^16^^,^^33^^,^^34^ The same papers were found in more than one database as well as in the manual search. No randomized clinical trials that evaluated the effectiveness of lapatinib as monotherapy for first-line or second-line treatment of advanced or metastasized breast cancer were found. Nor were any randomized clinical trials assessing the effectiveness of lapatinib in association with another drug for first-line treatment for metastasized breast cancer found. The search strategy and its results are contained in Table 1.

The clinical trial included in the systematic review evaluated 324 women with advanced breast cancer with HER-2 receptors (T4 primary tumor and stage IIIB or IIIC) or with metastasized cancer that had progressed subsequent to first-line treatment with anthracycline, taxane and trastuzumab.^16^ The cancer was evaluated according to the Response Evaluation Criteria in Solid Tumors (RECIST), modified to include lesions measuring 15 to 19 mm, with the ventricular ejection fraction within normal range as established by the institution and with the renal, hepatic and hematological systems functioning properly. Women with previous heart conditions, illnesses that could interrupt gastrointestinal absorption and patients who had ever received capecitabine as monotherapy before the beginning of this study were excluded. One group (n = 161) received capecitabine 2500 mg/m^2^/day orally on days 1 and 14 in a 21-day cycle and the other group (n = 163) received capecitabine 2000 mg/m^2^/day orally on days 1 and 14 every 3 weeks and lapatinib 1250 mg/day orally, continuously. The randomization was implemented in groups of six patients and, for the groups to be homogenous and comparable, they were stratified according to the cancer stage and the presence or absence of visceral disease.

The primary outcome was the “progression time”, defined as the length of time between randomization and the occurrence of disease progression or death due to breast cancer. The secondary outcomes were: length of survival free from disease progression (the length of time between randomization and disease progression or death from any cause), the total response rate, the clinical benefit rate (full or partial response or stabilization of the disease for at least six months) and safety. The patients were evaluated every six weeks over the first 24 weeks and, subsequently, every 12 weeks over the duration of the treatment. Intention to treat (effectiveness) analysis was carried out.

Compared with capecitabine alone, the association of lapatinib and capecitabine doubled the average length of time until disease progression, which was 8.4 months for the latter and 4.4 months for the group treated solely with capecitabine (hazard ratio = 0.49; 95% confidence interval, CI: 0.34-0.71; P < 0.001). In other words, the combined treatment reduced the risk of breast cancer progression by 50%, in relation to treatment solely with capecitabine. An increase in the length of time until disease progression was observed for all patients in the group that received the combined treatment. The secondary outcomes are shown in Table 2.

Four patients in the combined therapy group developed cerebral metastasis as the first progression site, compared with 11 patients in the group treated solely with capecitabine (P = 0.10). The frequency of adverse effects was similar in the two groups, with the exception of diarrhea, which occurred more frequently in the combined-treatment group (P < 0.001). The treatment discontinuation rate due to side effects was 13% in the combined-therapy group and 12% in the group treated solely with capecitabine. The most common adverse effects in both groups were diarrhea, hand-foot syndrome, nausea, vomiting, fatigue and rash. Asymptomatic reduction of the left ventricular ejection fraction ≥ 20% was observed in four patients in the combined-treatment group and in one patient in the single-treatment group, but there were no symptomatic cardiac events or cases of discontinuation of the treatment due primarily to cardiac function.

**Table 1.** Search strategies and results

Source	Search strategy	Number	RCT criteria selection
Cochrane Library	(lapatinib or tykerb or gw572016 or gw282974x or lapatinib and ditosylate)	16	1
PubMed	#1 (“lapatinib “[Substance Name]) OR (lapatinib) OR (GW 282974X) OR (GW-282974X) OR (GW282974X) OR (GW572016) OR (GW 572016) OR (GW-572016) OR (lapatinib ditosylate) OR (N-(3-chloro-4-(((3-fluorobenzyl)oxy)phenyl)-6-(5-(((2-methylsulfonyl)ethyl)amino)methyl) -2-furyl)-4-quinazolinamine) OR (Tykerb) #2 (“Breast Neoplasms”[Mesh]) OR (Breast Neoplasm*) OR (Neoplasm, Breast) OR (Breast Tumors) OR (Breast Tumor) OR (Tumor, Breast) OR (Neoplasms, Breast) OR (Tumors, Breast) OR (Breast Cancer) OR (Cancer, Breast) OR (Cancer of Breast) OR (Cancer of the Breast) OR (Mammary Carcinoma, Human) OR (Carcinoma, Human Mammary) OR (Carcinomas, Human Mammary) OR (Human Mammary Carcinomas) OR (Mammary Carcinomas, Human) OR (Human Mammary Carcinoma) OR (Mammary Neoplasms, Human) OR (Human Mammary Neoplasm) OR (Human Mammary Neoplasms) OR (Neoplasm, Human Mammary) OR (Neoplasms, Human Mammary) OR (Mammary Neoplasm, Human) #3 (#1) AND (#2)	157	1
EMBASE	Search: ‘lapatinib’/exp AND ‘breast cancer’/exp AND [embase]/lim. Mapped terms: ‘lapatinib’ mapped to ‘lapatinib’ , term is exploded ‘breast cancer’ mapped to ‘breast cancer’, term is exploded Limited to: embase Period: All Publication Years	424	1
Lilacs	(“lapatinib “[Substance Name]) OR (lapatinib) OR (GW 282974X) OR (GW-282974X) OR (GW282974X) OR (GW572016) OR (GW 572016) OR (GW-572016) OR (lapatinib ditosylate) OR (N-(3-chloro-4-(((3-fluorobenzyl)oxy)phenyl)-6-(5-(((2-methylsulfonyl)ethyl)amino)methyl) -2-furyl)-4-quinazolinamine) OR (Tykerb) [Palavras]	0	0
SCIRUS	((lapatinib) OR (GW572016) OR (GW 572016) OR (Tykerb)) AND (breast neoplasm)	23	1
Web of Science	#1 TS=(lapatinib) DocType=All document types; Language=All languages; Database=SCI-EXPANDED; Timespan=1945-2007 #2 TS=(tyberk) DocType=All document types; Language=All languages; Database=SCI-EXPANDED; Timespan=1945-2007 #3 TS=tykerb DocType=All document types; Language=All languages; Database=SCI-EXPANDED; Timespan=1945-2007 #4 TS=lapatinib ditosylate DocType=All document types; Language=All languages; Database=SCI-EXPANDED; Timespan=1945-2007 #5 TS=GW 572016 DocType=All document types; Language=All languages; Database=SCI-EXPANDED; Timespan=1945-2007 #6 TS=breast Neoplasm DocType=All document types; Language=All languages; Database=SCI-EXPANDED; Timespan=1945-2007 #7#5 OR #4 OR #3 OR #1DocType=All document types; Language=All languages; Database=SCI-EXPANDED; Timespan=1945-2007 #8 #7 AND #6DocType=All document types; Language=All languages; Database=SCI-EXPANDED; Timespan=1945-2007	2	0
Manual Search	--	67	3
Total		689	7*

* There were overlaps in the references, resulting in a total of three papers, which actually reported the data from the same clinical study. Of these papers, one was an original article, and the others were congress abstracts published in journal supplements. RCT = randomized clinical trial.

**Table 2.** Outcomes from intention-to-treat (effectiveness) analysis^16^

Outcomes	Lapatinib + capecitabine (n=163)	Capecitabine alone (n=161)	Risk ratio (95% CI)	P-value
Average length of time until disease progression (months)	8.4	4.4	0.49 (0.34-0.71)	< 0.001^*^
Average length of survival free from disease progression (months)	8.4	4.1	0.47 (0.33-0.67)	< 0.001^*^
Total response rate % (95% CI)	22 (16-29)	14 (9-21)	-	0.09^†^
Full response n (%)	1 (<1)	0 (0)	-	-
Partial response n (%)	35 (21)	23 (14)	-	-
Patients with clinical benefit n (%)	44 (27)	29 (18)	-	-
Deaths n (%)	36 (22)	35 (22)	-	-

CI = confidence interval. ^*^log-rank test; ^†^ Fisher’s exact test.

## DISCUSSION

Considering that only one clinical trial evaluating the effectiveness of lapatinib for treating advanced or metastasized breast cancer was found and selected, and considering that this study only dealt with the use of lapatinib for second-line treatment and in association with capecitabine, there are limitations on the results from this systematic review, despite their relevance.

With regard to methodological evaluation, the study that was reviewed was given a rating of B on the scale set out in the Cochrane Collaboration Handbook and a rating of 3 on the Jadad scale, since it demonstrated an adequate randomization technique, was double blind and described losses and exclusions.^31^^,^^32^ Nevertheless, the study presented a risk of breakage of the allocation concealment, since the method through which the treatments were administered was not considered identical between the two groups. This was because of a lack of reporting on whether the group treated solely with capecitabine also continuously received placebo. The foregoing results allowed us to classify the study as having moderate risk of bias.^31^^,^^32^ According to the level-of-evidence classification established by the Oxford Centre for Evidence-Based Medicine, the results from this clinical study support the use of lapatinib in association with capecitabine, at the doses prescribed and for this specific group of patients with advanced or metastasized breast cancer, with a level of evidence of 1b and a grade of recommendation of A.^35^ The study was financed and conducted by GlaxoSmithKline.

The published results from this trial correspond to an internal analysis (n = 324) on a study that intended to include 528 patients (statistical power = 90%, a = 0.05) and which was conducted after 114 instances of cancer progression. Since this analysis demonstrated that the addition of lapatinib to capecitabine was associated with a reduction in the risk of disease progression by 51%, the safety monitoring committee recommended that the study should be closed and that a report containing this information should be drafted.

Based on updated data from this study, in March 2007 the Swiss government and the United States (US) Food and Drug Administration (FDA) approved the use of lapatinib in combination with capecitabine for treatment of metastasized or advanced breast cancer with HER-2 receptors, for patients who had received anthracycline, taxane and trastuzumab as first-line treatment.^30^^,^^36^ The updated results demonstrated that there was a significant increase in the average length of time until cancer progression in the combined-treatment group, from 18.6 to 27.1 weeks (HR = 0.57; 95% CI 95%: 0.43-0.77; P = 0.00013).^36^ The total response rate also remained high in the combined-treatment group: 24% (95% CI: 18%-30.3%) versus 14% (95% CI: 9.5%-19.5%).^36^

No randomized clinical trials evaluating the effectiveness of lapatinib in any of the following situations were found: a) as the sole first-line or second-line treatment for advanced or metastasized breast cancer; b) as part of a combined treatment for the first-line or second-line treatment of advanced or metastasized breast cancer; c) as part of a combined treatment for first-line treatment of advanced or metastasized breast cancer; or d) as part of a combined treatment with a drug other than capecitabine for second-line treatment of advanced or metastasized breast cancer. Therefore, Table 3 includes data from phase II and III clinical studies that have already been implemented or are in progress, with objectives that fit within the foregoing list.^37^^,^^38^^,^^39^^,^^40^^,^^41^^,^^42^^,^^43^^,^^44^^,^^45^^,^^46^^,^^47^^,^^48^^,^^49^^,^^50^^,^^51^^,^^52^^,^^53^

**Table 3.** Phase II and III studies on lapatinib for treatment of advanced or metastasized cancer

Study	Intervention	Phase	Primary outcome	Full response (%)	Partial response (%)	Stabilized disease (%)	IPT (months)
First-line treatment							
EGF20009^37^	lapatinib 1 dose/day versus 2 doses/day	II	GRR	0	35	35	NR
EGF30001^38^	paclitaxel/lapatinib versus paclitaxel/placebo	III	IPT, GRR survival biomarker	In progress			
EGF104383^39^	paclitaxel/trastuzumab/lapatinib versus paclitaxel/trastuzumab/placebo	III	IPT, GRR survival biomarker	in progress			
EGF30008^40^	letrozole/lapatinib versus letrozole/placebo	III	IPT, GRR survival biomarker	in progress			
EGF104353^41^	paclitaxel/lapatinib versus paclitaxel/placebo	III	IPT, GRR survival biomarker	in progress			
(Refractory) second-line or third-line treatment							
EGF20002^42^^,^^43^	Lapatinib	II	GRR safety	8*	-	14	22%^†^
EGF20008^43^^,^^44^	Lapatinib	II	GRR safety				
cohort A (HER-2 +)				4*	-	9^+^	13%^†^
cohort B (HER-2 -)				0*	-	1	
EGF104900^45^	trastuzumab/lapatinib versus lapatinib	III	GRR SFP	in progress		
EGF105084^46^	lapatinib (cerebral metastasis)	II	GRR	16.3% of the patients show a 20% volume reduction in the cerebral lesion		
NCI-CTEP 6969 ^47^	lapatinib (cerebral metastasis)	II	GRR in the CNS	0	5^‡^	NR	3.2
Inflammatory breast cancer							
EGF103900^48^	Lapatinib	II	GRR				NR
cohort A				0	62	21	
cohort B				0	8	17	
EGF102580^49^	Lapatinib + paclitaxel versus (neoadjuvant)	II	NR	in progress			
Adjuvant treatment							
ALTTO^50^	trastuzumab one year versus lapatinib one year versus trastuzumab/lapatinib one year versus trastuzumab three months ®lapatinib nine months	III	survival IPT, GRR safety	in progress			
TEACH^51^^,^^52^	lapatinib one year versus placebo	III	survival SFI, QOL recurrence CNS	in progress			
Neoadjuvant treatment							
NeoALTTO^53^	lapatinib/paclitaxel versus trastuzumab/paclitaxel versus lapatinib/trastuzumab/paclitaxel	III	GRR, IPT safety tolerability survival	in progress			

ALTTO = Adjuvant Lapatinib and/or Trastuzumab Treatment Optimization; NeoALTTO = Neoadjuvant Lapatinib and/or Trastuzumab Treatment Optimization; NR = not reported; QOL = quality of life; SFI = survival free from illness; SFP = survival free from progression; CNS = central nervous system; TEACH = Tykerb Evaluation After Chemotherapy; IPT = illness progression time; GRR = general response rate; EGF = epidermal growth factor.

* general response, including full and partial response; ^†^ 16 weeks of survival free from cancer progression; ^‡^CNS response/cerebral metastases response.

## CONCLUSIONS

The combination of lapatinib and capecitabine was more effective than capecitabine alone for reducing the risk of cancer progression in women with metastasized or advanced breast cancer with overexpression of HER-2 receptors and who had received first-line treatment with anthracycline, taxane and trastuzumab.

Lapatinib seems to be an adequate adjuvant treatment for HER-2-positive breast cancer, since it demonstrates effectiveness in cases of advanced and metastasized cancer, appears to have few serious side effects and may be associated with reduction of the incidence of cerebral metastasis, in addition to its easy oral administration once a day.

Randomized clinical trials need to be implemented, with the objective of assessing the effectiveness of lapatinib as monotherapy for first-line or second-line treatment of advanced breast cancer, as well the effectiveness of lapatinib in associations as the primary treatment. An economic study with a cost effectiveness evaluation is also necessary, in order to incorporate this new technology into clinical practice.
